# Fault Diagnosis of Rotating Machinery Using Supervised Machine Learning Algorithms with Integrated Data-Driven and Physics-Informed Feature Sets

**DOI:** 10.3390/s26061876

**Published:** 2026-03-17

**Authors:** Anastasija Angjusheva Ignjatovska, Zlatko Petreski, Viktor Gavriloski, Dejan Shishkovski, Simona Domazetovska Markovska, Maja Anachkova, Damjan Pecioski

**Affiliations:** Faculty of Mechanical Engineering Skopje, Ss. Cyril and Methodius University in Skopje, 1000 Skopje, North Macedonia; zlatko.petreski@mf.edu.mk (Z.P.); viktor.gavriloski@mf.edu.mk (V.G.); dejan.shishkovski@mf.edu.mk (D.S.); simona.domazetovska@mf.edu.mk (S.D.M.); maja.anachkova@mf.edu.mk (M.A.); damjan.pecioski@mf.edu.mk (D.P.)

**Keywords:** fault diagnosis, rotating machinery, vibration analysis, supervised machine learning, physics-informed features, data-driven features

## Abstract

This study proposes a supervised machine learning framework for vibration-based fault diagnosis of rotating machinery using integrated data-driven and physics-informed feature sets. A dataset acquired under variable load and multiple operating conditions was used for model training. Parallel signal processing techniques were applied to capture fault-related information across multiple frequency bands including time-domain analysis, frequency-domain analysis, baseband analysis, and envelope analysis. From the corresponding signal representations, statistical, spectral, and physics-based features associated with characteristic fault frequencies were extracted and combined into integrated feature sets. The diagnostic performance of models trained using purely data-driven features was systematically compared with models incorporating integrated data-driven and physics-informed features. Support Vector Machine, Random Forests, Gradient Boosting, and an ensemble classifier were evaluated using accuracy, precision, recall, and F1-score metrics. The proposed framework employs a two-layer classification strategy, where the first layer performs multiclass fault identification, while the second layer evaluates the presence of imbalance as a coexisting fault. In addition, the influence of different feature groups as well as individual measurement axes and their combinations on diagnostic performance were analyzed. Validation using a new dataset measured in laboratory conditions confirmed the robustness and generalization capability of the proposed diagnostic framework.

## 1. Introduction

Traditional vibration-based diagnostic methods rely on expert interpretation of time-domain indicators and frequency spectra, which limits their practical use in large-scale industrial monitoring applications [1,2,3,4]. As industrial machinery operates under variable load and speed conditions, diagnostic approaches must remain reliable across different machines and measurement setups. As a result, increasing attention has been directed toward automated fault diagnosis methods that can generalize beyond the conditions represented in the training data [2,5,6].

In this context, machine learning techniques have been widely adopted to enable the data-driven modeling of relationships between vibration signals and machine health conditions. Among these approaches, supervised machine learning has emerged as an effective solution for automating vibration-based fault diagnosis by learning mappings between signal characteristics and predefined fault classes [7,8,9]. These methods typically involve lower model complexity, require fewer training samples than more complex data-driven approaches, and offer transparency in feature interpretation, which is particularly relevant for industrial diagnostic applications [10,11,12,13,14,15].

In many studies, diagnostic models are developed using features extracted directly from vibration signals in the time and frequency domains, including statistical indicators and global spectral descriptors [7,8,16,17,18]. To enhance diagnostic performance, several works have combined statistical time-domain features with frequency-domain features or envelope-based representations, reporting improved classification accuracy for rolling bearing faults under varying load conditions [19,20,21,22].

Beyond these approaches, feature sets that integrate time-domain, frequency-domain, and time–frequency information have been investigated to capture complementary fault-related characteristics and improve fault discrimination [23,24,25,26]. Time–frequency representations such as wavelet transforms, empirical mode decomposition, and related adaptive signal analysis techniques have been employed to extract features sensitive to nonstationary and transient vibration components [27,28,29]. More recently, several studies have explored the combination of multiple feature groups derived from different signal representations to improve robustness under variable operating conditions and to enhance fault separability in complex diagnostic scenarios [30,31,32,33].

While such approaches can achieve high classification accuracy under controlled experimental conditions, their performance is often sensitive to changes in operating conditions, sensor placement, and machine configuration [32,34,35,36].

Consequently, diagnostic reliability may degrade when different fault types produce similar vibration patterns or when test conditions differ from those used during model training.

A key limitation of purely data-driven feature sets is that they describe the overall signal behavior but do not emphasize vibration components that are directly linked to specific faults. As a result, fault-related information concentrated in narrow frequency bands may be obscured by other vibration sources, noise, or coexisting fault effects [37,38,39], particularly under varying load conditions. Recent research has demonstrated that incorporating physics-informed features into machine learning-based diagnostic models can improve robustness, interpretability, and generalization capability. Physics-guided feature engineering has been shown to enhance classification performance across fault conditions and speed regimes when compared with purely statistical descriptors [40,41,42,43] and deep learning approaches [44]. Similarly, classical ML methods which use feature ranking and selection continue to achieve high accuracy with reduced complexity, reaffirming the relevance of engineered features for practical diagnostics [45].

Despite these advances, systematic comparisons between purely data-driven feature sets and integrated data-driven and physics-informed feature sets within the same supervised learning framework are still scarce. Moreover, the coexistence of multiple faults, such as imbalance occurring alongside dominant bearing defects, a common scenario in real industrial machinery, has received relatively limited attention in existing diagnostic studies [46,47].

From a physical standpoint, this challenge is closely related to the fact that faults in rotating machinery arise from distinct mechanical mechanisms that manifest across different frequency ranges. Faults such as imbalance and misalignment predominantly excite low-frequency vibration components related to rotational speed and its harmonics [48,49,50], whereas rolling bearing defects generate high-frequency, impulsive responses that are typically revealed through demodulation techniques [51,52,53,54]. The simultaneous presence of low-frequency and high-frequency fault signatures creates a significant challenge for classification, as a single signal preprocessing or representation is often insufficient to reliably discriminate between these fault types. Signal processing techniques such as baseband analysis, spectral kurtosis, and envelope analysis are therefore important for isolating physically meaningful fault-related components and enhancing sensitivity to localized bearing damage.

In addition, many existing studies restrict evaluation to a single classifier, a single signal representation, or a fixed sensor orientation, which can limit the general applicability of the reported results [55,56,57,58]. The influence of measurement axis selection and multi-axis feature integration on diagnostic performance is also often treated only marginally, despite its practical relevance in industrial condition monitoring systems.

In recent years, deep learning techniques such as convolutional neural networks (CNNs), recurrent neural networks (RNNs), and hybrid architectures have become increasingly popular for the fault diagnosis of rotating machinery using vibration and other sensor signals. These methods have been shown to automatically learn complex feature representations from raw sensor data and achieve high classification performance in many benchmark scenarios [59]. Recent studies have reported strong diagnostic performance using CNN-based spectrogram analysis, hybrid CNN–RNN models, and lightweight transformer architectures for bearing fault classification under controlled experimental conditions [60,61,62].

While deep learning models have demonstrated strong performance on large benchmark datasets, their practical deployment in industrial monitoring environments may be constrained by data availability, computational requirements, and limited interpretability. In addition, the learned internal representations are often difficult to interpret in terms of explicit physical fault mechanisms. In many practical industrial applications, datasets are moderate in size, operating conditions vary across machines, and diagnostic systems must support computational efficiency and explainable decision-making. Under such constraints, feature-engineered supervised learning approaches remain highly relevant, particularly when combined with physics-informed descriptors that explicitly encode characteristic fault frequencies and harmonic energy distributions associated with known mechanical defects. Accordingly, the objective of the present study is not to compare model architectures, but to systematically investigate the contribution of physics-informed feature integration within a supervised learning framework, with emphasis on robustness, interpretability, and practical applicability in industrial monitoring environments.

In this context, the present study developed a supervised machine learning-based diagnostic framework that systematically integrates data-driven and physics-informed feature sets for the vibration-based fault diagnosis of rotating machinery. Multiple signal representations were processed in parallel to extract complementary information from the time-domain, frequency-domain, baseband, and envelope analyses. The extracted features were combined and evaluated using several supervised learning algorithms, including Support Vector Machines, Random Forests, Gradient Boosting, and an ensemble classifier, allowing for a consistent comparison of diagnostic performance across different modeling strategies.

A two-layer classification strategy was introduced, in which the first layer identifies the dominant fault type, while the second layer assesses the presence of imbalance as a coexisting fault, reflecting common industrial fault scenarios. In addition, the influence of individual measurement axes and their combinations on diagnostic performance was investigated. The proposed approach was validated using an independent laboratory dataset to assess robustness and generalization capability under previously unseen measurement conditions.

**Figure 1:**
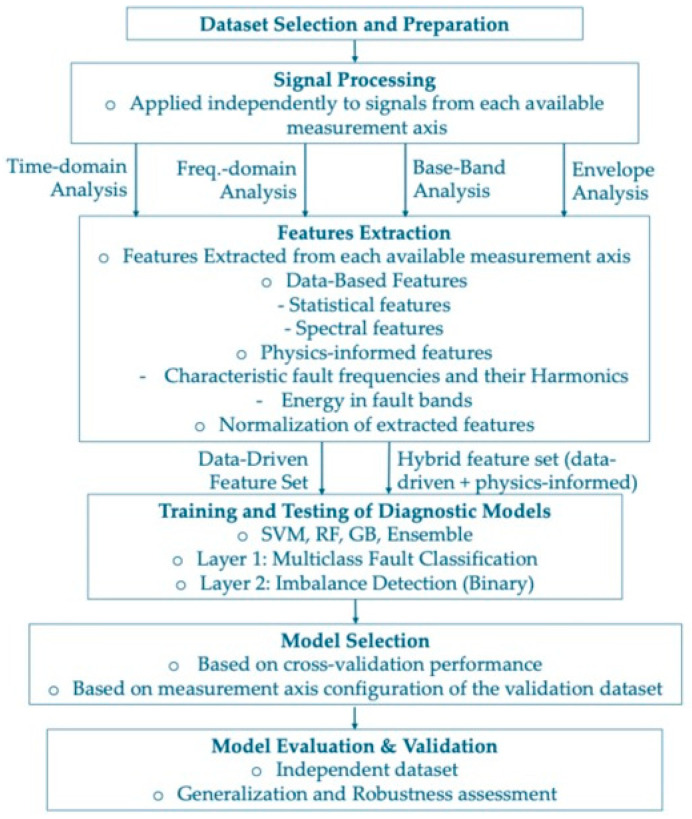
Complete overview of the proposed diagnostic framework.

Figure 1 illustrates the proposed diagnostic framework in which signal processing and feature extraction were applied independently to each available measurement axis. Two feature sets (data-driven and hybrid) were used to train supervised classifiers within a two-layer classification scheme. The final model was selected based on cross-validation performance and measurement axis configuration and validated using an independent dataset.

## 2. Dataset Description and Preparation

The performance of vibration-based fault diagnosis machine learning models strongly depends on the quality and consistency of the dataset used for training. Therefore, the dataset must reflect realistic operating conditions, provide reliable fault annotations, and contain sufficient information to capture characteristic fault-related vibration components.

In this study, the open-source Machinery Fault Database was selected due to its structured fault scenarios, varying load conditions, and wide use in rotating machinery diagnostics. The dataset was acquired using a SpectraQuest Machinery Fault Simulator [63] consisting of an electric motor, shaft, rotors, and rolling bearings. The shaft is supported by two rolling bearings located on opposite sides of the rotor, referred to as the underhang bearing (located between the motor and the rotor) and the overhang bearing (located beyond the rotor), following the terminology of the SpectraQuest test rig. The technical specifications of the used rolling bearings in the simulator are shown in Table 1.

Vibration signals were recorded under controlled laboratory conditions for several fault types, including normal operation, imbalance, misalignment, and rolling bearing defects. In total, ten machine states were considered: normal condition, imbalance, horizontal misalignment, vertical misalignment, faulty outer ring, faulty ball, and faulty cage of the underhang bearing, as well as a faulty outer ring, faulty ball, and faulty cage of the overhang bearing. Vibration measurements were collected using accelerometers mounted on different bearing locations and along multiple measurement axes, enabling the investigation of axis-dependent diagnostic performance.

To ensure consistency across all operating conditions and sensor configurations, the vibration signals were uniformly resampled from the original sampling frequency of 50 kHz to a common value of 5 kHz. This choice was dictated by the frequency bandwidth of the triaxial accelerometer mounted using a magnetic base, which provides reliable measurements up to approximately 5 kHz. The selected sampling frequency remained sufficient to capture all characteristic fault frequencies and their relevant harmonics for the bearing geometry and rotational speed range considered in the dataset. An anti-aliasing filter was applied prior to downsampling to prevent spectral distortion.

**Figure 2:**
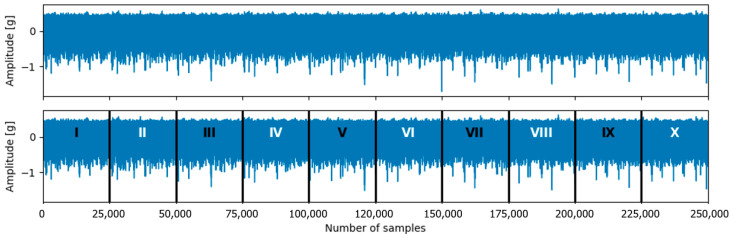
Illustration of the proposed balancing methodology applied to normal-condition signals in the MaFaulda dataset.

The original dataset exhibited a strong class imbalance, particularly for the normal operating condition. To address this issue, a balancing method was proposed, which is illustrated in Figure 2. The method performs a signal segmentation strategy applied only to normal-condition signals, increasing the number of available samples without introducing artificial fault information.

After downsampling, each 5 s vibration signal contained 25,000 samples. Each normal-state signal was divided into ten non-overlapping segments of equal length. Each segment contained 2500 samples, corresponding to a duration of 0.5 s. To avoid excessive replication and reduce the risk of overfitting, only every second segment was retained, resulting in five segments per original signal. Consequently, the number of normal-state samples increased from 49 to 245. After balancing, the final dataset contained 2147 samples across ten classes, with the normal class representing approximately 11.4% of the dataset. The number of samples for all faulty classes remained unchanged.

The segmentation intervals were strictly non-overlapping, meaning that no sliding-window or overlapping segmentation was applied. The segmentation procedure was completed before feature extraction and model training and testing. In the subsequent evaluation stage (Section 5), a 10-fold cross-validation technique was applied to the final balanced dataset. Since segmentation was performed before the data were partitioned into training and testing folds, non-overlapping segments originating from the same original 5 s signal may be distributed across different folds. However, as these segments corresponded to distinct temporal intervals and did not overlap, this did not introduce identical data duplication.

The proposed balancing method improved class balance while preserving the physical characteristics of the vibration signals.

Following the balancing procedure, the dataset was prepared for feature extraction and physics-informed analysis.

The shaft rotational frequency $f_{r}$ was determined using the tachometer signal included in the dataset and used for computing characteristic fault frequencies required for physics-informed feature extraction. Following these steps, the prepared dataset provided a consistent and physically meaningful basis for signal processing, feature extraction, and supervised fault classification.

## 3. Signal Processing and Feature Extraction

Following the dataset preparation described in Section 2, signal processing and feature extraction were performed to obtain compact descriptors that emphasize vibration components associated with machine faults. Multiple signal processing methodologies were applied in parallel, as summarized in Figure 1, to capture fault-related information distributed across different frequency bands.

Accordingly, several complementary signal representations were obtained after applying the chosen processing methodologies: (i) the time-domain vibration signal, (ii) the frequency spectrum, (iii) a low-frequency baseband representation highlighting rotational components, and (iv) an envelope-based representation highlighting impulsive bearing responses. Due to the diverse physical nature of the considered faults, each feature group was extracted from the signal representation that best highlighted the corresponding fault mechanisms. This strategy ensured high sensitivity to different defect types while maintaining robustness across varying operating conditions.

Signal processing and feature extraction were performed independently for each available measurement axis. Let $x_{a}\left(t\right)$ denote the vibration signal measured along axis a. For each axis, the same processing pipeline is applied to generate the corresponding feature vector $f_{a}$. Axis configurations are then formed by using either a single axis ($f_{a}$) or by concatenating multiple axes ($\left[f_{a_{1}},\;f_{a_{2}},\;...\right]$). This design enabled a systematic comparison of single-axis and multi-axis configurations under the same learning and validation protocol.

The extracted features were organized into two principal sets: a data-driven feature set and a hybrid feature set. In the following, the vibration signals are denoted as rad1, tg1, and ax1 for the radial, tangential, and axial directions measured at the overhang bearing, and as rad2, tg2, and ax2 for the corresponding directions measured at the underhang bearing, respectively.

### 3.1. Data-Driven Feature Extraction

The data-driven feature set consisted of time-domain statistical features and frequency-domain statistical and spectral features.

#### 3.1.1. Time-Domain Features

Time-domain features are statistical indicators extracted directly from the prepared vibration signal $x_{a}(t)$ and describe the amplitude distribution and impulsiveness of the waveform. These features provide compact descriptors of signal energy and shape and are widely used as baseline inputs for vibration-based fault diagnosis.

The following time-domain statistical features were extracted per axis: Root Mean Square (RMS), Standard Deviation (Std), Kurtosis, Skewness, Peak Value, Crest Factor, Impulse Factor, Margin Factor, Shape Factor, Entropy, and Root Mean Square of Vibration Velocity RMS (v_RMS_).

Table 2 provides an example of the computed time-domain statistical feature values for the three measurement axes on both bearings, for a case involving a rolling element (ball) defect on the overhang bearing combined with an imbalance fault of 6 g at a shaft rotational speed of 39.73 Hz.

#### 3.1.2. Frequency-Domain Features

Frequency-domain features were obtained by transforming the prepared vibration signal $x_{a}(t)$ into the frequency domain using the Fast Fourier Transform (FFT). These features describe the global distribution of spectral energy and enable the detection of changes in frequency content associated with variations in machine operating condition and fault presence.

The following frequency-domain statistical features were extracted from the amplitude spectrum of prepared signal per axis: Frequency Center (FC), Root Mean Square Frequency (RMSF), and Root Variance Frequency (RVF). In addition, frequency-domain spectral features were extracted with respect to the tachometer reference signal. These included the phase angle φ and its sine and cosine components per axis, which allowed us to represent the phase information in a continuous and machine-learning-compatible form.

Table 3 presents an example of the computed frequency-domain statistical feature values for the three measurement axes on both bearings, under the condition of a ball defect in the outer bearing combined with an imbalance of 6 g and a shaft rotational speed of 39.73 Hz. Table 4 presents an example of the corresponding spectral feature values for the same operating condition and fault combination.

While the data-driven features describe the global statistical and spectral properties of the vibration signals, they do not explicitly encode fault-specific physical mechanisms. Therefore, a hybrid feature set combines data-driven features with physics-informed features derived from baseband and envelope analysis, in order to enhance interpretability and explicitly exploit fault-mechanism knowledge.

### 3.2. Physics-Informed Feature Extraction

In addition to the data-driven feature set, a physics-informed feature set was constructed using baseband and envelope analysis in order to explicitly incorporate fault-mechanism knowledge. The shaft rotational frequency $f_{r}$ was obtained from the tachometer signal using the explained methodology in [64,65,66] and used to locate rotational harmonics and bearing characteristic frequencies in the frequency domain. Physics-informed features include:

(i) Rotational harmonic energy features (baseband spectrum).

Baseband analysis is employed to extract energy-based indicators associated with imbalance and misalignment faults. Specifically, the energy contained at the first harmonic $E_{1X}$ and the misalignment index ${MIS}_{index}$ are computed for each measurement axis. The misalignment index is defined as:

(1) $${MIS}_{index}=\frac{E_{2X}+0.5\;E_{3X}}{E_{1X}+\varepsilon }\;$$

where $E_{2X}$ and $E_{3X}$ denote the energy contained at the second and third rotational harmonics, respectively, and ε is a small constant introduced to avoid division by zero.

(ii) Energy ratios at bearing characteristic frequencies (envelope spectrum).

Envelope analysis is employed to extract energy-based indicators associated with rolling bearing defects. The energy content around the Ball Pass Frequency of the Outer race (BPFO), Ball Spin Frequency (BSF), and Fundamental Train Frequency (FTF) is computed from the envelope spectrum. The corresponding energy ratios are defined as:

(2) $$BPFO_{ratio}=\frac{E_{BPFO}}{E_{BPFO}+E_{BSF}+E_{FTF}}\;$$

(3) $${BSF}_{ratio}=\frac{E_{BSF}}{E_{BPFO}+E_{BSF}+E_{FTF}}\;$$

(4) $${FTF}_{ratio}=\frac{E_{FTF}}{E_{BPFO}+E_{BSF}+E_{FTF}}\;$$

where $E_{BPFO}$, $E_{BSF}$, and $E_{FTF}$ denote the energy contained in narrow frequency bands centered at the respective characteristic frequencies.

(iii) Harmonic energy components of bearing characteristic frequencies.

To capture higher-order repetitions of fault-induced modulation and to account for increasing fault severity, harmonic components of the bearing characteristic frequencies are additionally extracted. These include:

○ the energy contained at the first four harmonics of BPFO

$$\left(E_{BPFO\_1X},\;E_{BPFO\_2X},\;E_{BPFO\_3X},\;E_{BPFO\_4X}\right),$$

○ the energy contained at the first two harmonics of BSF

$$\left(E_{BSF\_1X},\;E_{BSF\_2X}\right),\mathrm{and}$$

○ the energy contained at the first four harmonics of FTF

$$\left(E_{FTF\_1X},\;E_{FTF\_2X},\;E_{FTF\_3X},\;E_{FTF\_4X}\right).$$

These physics-informed features provide interpretable descriptors linked to known fault mechanisms and complement the purely statistical features used in the data-driven feature set.

#### 3.2.1. Baseband Analysis (Rotational Harmonics)

To characterize faults that predominantly affect the low-frequency vibration content, such as imbalance and misalignment, baseband analysis was applied to isolate and analyze the rotational harmonics of the shaft.

A fourth-order low-pass Butterworth filter with a cut-off frequency of 250 Hz was applied to the vibration signal to retain the first four harmonics (1–4×) of the maximum rotational speed considered in the dataset. Components at higher frequencies, mainly related to bearing faults and structural resonances, were suppressed.

Prior to transformation into the frequency domain, the filtered signal was windowed using a Hann window to reduce spectral leakage caused by non-periodic signal segments. The windowed signal was then transformed using Fast Fourier Transform (FFT), yielding the baseband spectrum dominated by the shaft rotational frequency and its low-order harmonics.

From the resulting baseband spectrum, physics-informed features were extracted for each measurement axis, including:

○ the energy contained around the first, second, and third rotational harmonics (1×, 2×, and 3×), and
○ the misalignment index defined in Equation (1).

**Figure 3:**
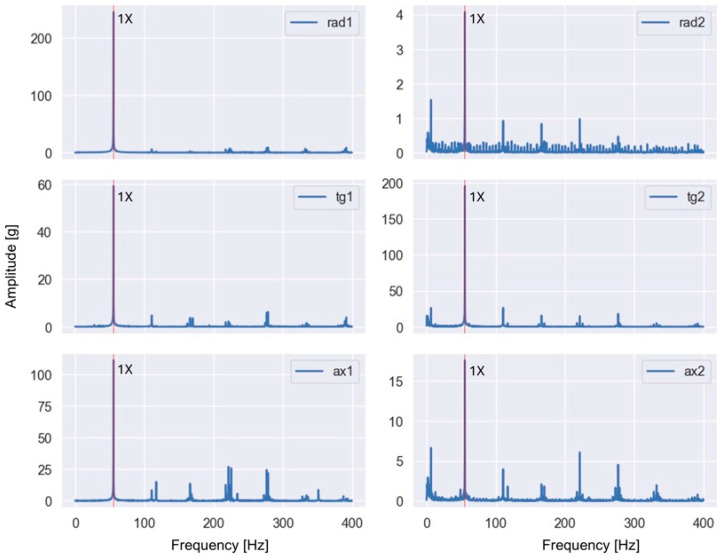
Example baseband spectrum of a vibration signal measured in the presence of imbalance (35 g) at a rotational frequency of 55.40 Hz (1× harmonic is dominant and highlighted with red).

**Figure 4:**
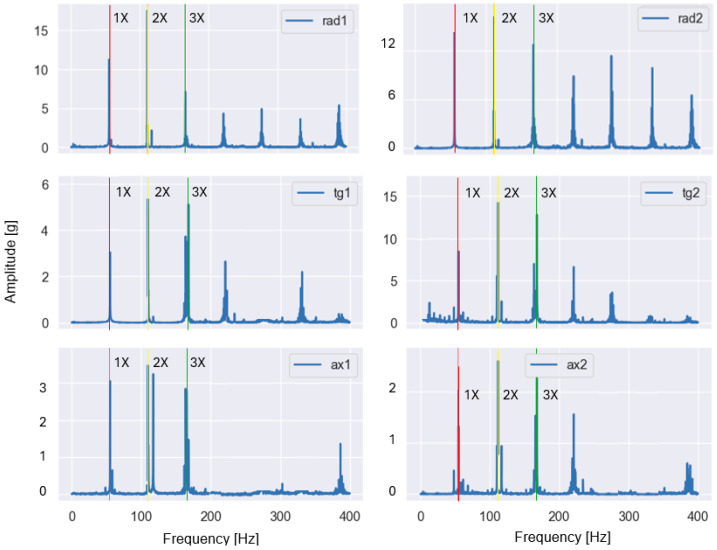
Baseband spectrum of a vibration signal measured in the presence of misalignment (1.90 mm) at a rotational frequency of 56.73 Hz (2× is dominant, 1× is highlighted with red, 2× with yellow, 3× with green).

These features are particularly sensitive to imbalance and misalignment, which are known to produce pronounced vibration components at the fundamental rotational frequency and its harmonics. Representative examples of the baseband spectra obtained after low-pass filtering and FFT are shown in Figure 3 and Figure 4 for selected fault conditions.

For the imbalance case (Figure 3), the dominant peak occurred at the fundamental rotational frequency (1×), whereas for the misalignment case (Figure 4), increased energy was observed at the second harmonic (2×). The observed spectral patterns in Figure 3 and Figure 4 were consistent with the theoretical fault signatures of imbalance and misalignment, confirming the validity of the applied baseband processing and the extracted harmonic-energy features.

#### 3.2.2. Envelope Analysis (Bearing Fault Demodulation)

Rolling bearing defects generate impulsive excitations due to repeated impacts between rolling elements and localized fault surfaces. These impulses excite structural resonances of the bearing–housing–sensor system, resulting in amplitude-modulated high-frequency vibration components. Consequently, characteristic bearing fault frequencies are not directly visible in the conventional amplitude spectrum but are encoded in the envelope of the high-frequency response.

Envelope analysis was therefore employed to demodulate the vibration signal and reveal bearing characteristic frequencies in the envelope spectrum. A fourth-order band-pass Butterworth filter with cut-off frequencies of 300 Hz and 2000 Hz was first applied to suppress low-frequency rotational components and isolate the resonance band excited by bearing impacts. The selection of the band-pass interval was based on both physical considerations and dataset-specific operating conditions.

The lower cut-off frequency of 300 Hz was determined from the maximum rotational speed present in the MaFaulDa dataset (approximately 60 Hz). Rotational harmonics associated with imbalance and misalignment were expected up to approximately the fourth harmonic (4 × 60 Hz ≈ 240 Hz). Therefore, selecting a lower bound of 300 Hz ensured the suppression of rotational components while retaining higher-frequency resonance bands excited by bearing fault impacts.

The upper cut-off frequency of 2000 Hz was selected considering both the resampled signal bandwidth (5 kHz sampling frequency; Nyquist frequency 2500 Hz) and the structural resonance characteristics of the bearing–housing system, which typically lie within the mid-frequency range (approximately 1–3 kHz). For the considered rotational speed range (11.7–60 Hz), the characteristic bearing fault frequencies and their significant harmonics remained well below 1000 Hz. For example, at the maximum shaft speed of 60 Hz, the fourth harmonic of BPFO and the second harmonic of BSF remained below 1000 Hz, ensuring that all diagnostically relevant envelope components were fully contained within the selected band.

Therefore, the chosen interval effectively isolates the resonance region excited by impact events while avoiding contamination from low-frequency rotational dynamics and unnecessary high-frequency noise.

It should be noted that this frequency range is dataset-dependent. For rotating machinery operating at significantly higher rotational speeds or exhibiting structural resonance outside this interval, the band-pass limits should be adjusted based on shaft speed, bearing geometry, and experimentally identified resonance characteristics.

The filtered signal was then processed using the Hilbert transform to obtain the analytic signal, and its magnitude was taken as the signal envelope. To remove residual high-frequency noise, the envelope was subsequently low-pass filtered using a fourth-order Butterworth filter with a cut-off frequency of 800 Hz. Prior to transformation into the frequency domain, the envelope signal was windowed using a Hann window to reduce spectral leakage and then transformed using Fast Fourier Transform (FFT), yielding the envelope spectrum.

Using the theoretical relations for calculating characteristic fault frequencies shown in Equation (5) to Equation (7) [67,68] and technical specifications of the rolling bearings used in the SpectraQuest simulator shown in Table 1, the values of the characteristic frequencies of the bearing defects including the Ball Pass Frequency of the Outer race (BPFO), Ball Spin Frequency (BSF), and Fundamental Train Frequency (FTF) were calculated as functions of the rotational frequency (Table 5).

Ball Pass Frequency Outer Race (BPFO)

(5) $$BPFO=\frac{N}{2}\cdot \left(1-\frac{D_{B}}{D_{P}}\cdot cos\varphi \right)\cdot f_{r}=N\cdot FTF$$

Ball Spin Frequency (BSF)

(6) $$BSF=\frac{D_{P}}{2\cdot D_{B}}\cdot \left(1-{\left(\frac{D_{B}}{D_{P}}\cdot cos\varphi \right)}^{2}\right)\cdot f_{r}$$

Fundamental Train Frequency (FTF)

(7) $$FTF=\frac{1}{2}\cdot \left(1-\frac{D_{B}}{D_{P}}\cdot cos\varphi \right)\cdot f_{r}$$

From the envelope spectrum, physics-informed features were extracted for each measurement axis, including:

(i) energy ratios around BPFO, BSF, and FTF components (Equations (2)–(4)), and
(ii) harmonic energy components at the first four harmonics of BPFO, the first two harmonics of BSF, and the first four harmonics of FTF.

These features capture the periodicity and severity of bearing fault-induced impacts and provide physically interpretable indicators of localized defects.

**Figure 5:**
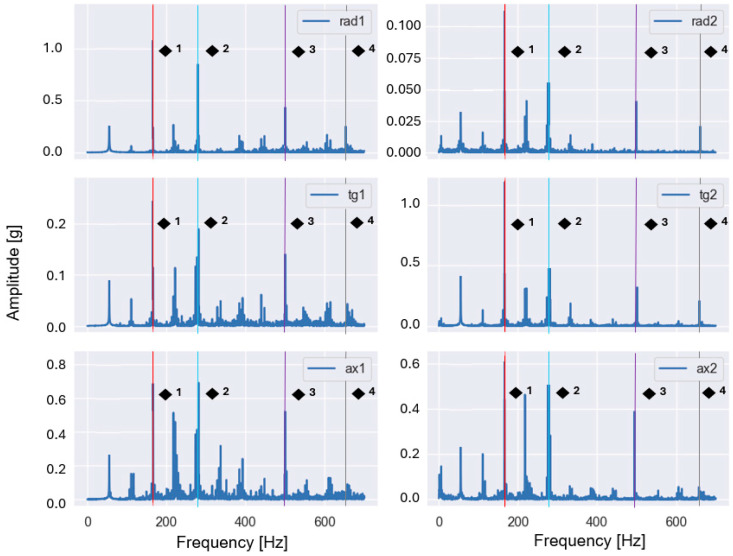
Envelope spectrum of a vibration signal measured in the presence of an outer race defect and imbalance (20 g) at a rotational frequency of 55.40 Hz (BPFO and its 2×, 3×, 4× harmonics are highlighted in red (◆^1^), blue (◆^2^), purple (◆^3^), and grey (◆^4^).

**Figure 6:**
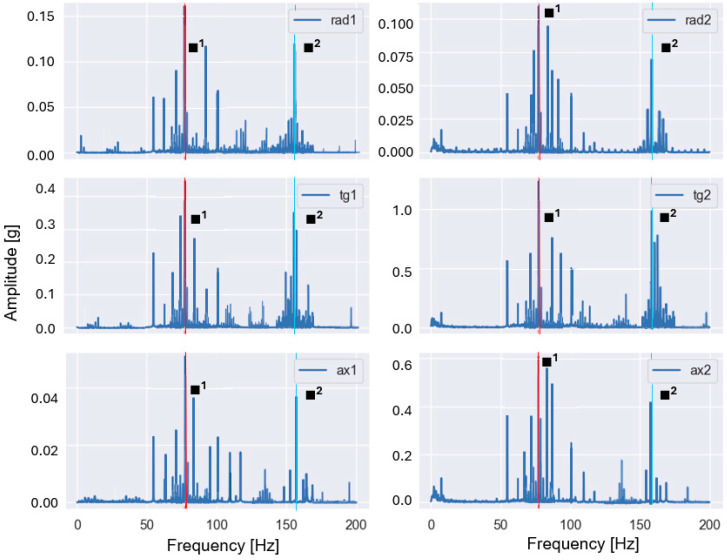
Envelope spectrum of a vibration signal measured in the presence of a rolling element defect and imbalance (20 g) at a rotational frequency of 54.80 Hz (BSF and its 2× harmonic are highlighted in red (■^1^) and blue (■^2^)).

Representative envelope spectra obtained after band-pass filtering, Hilbert demodulation, and FFT are shown in Figure 5 and Figure 6 for selected fault conditions.

The characteristic frequencies associated with rolling bearing defects can be calculated from the bearing geometry and the shaft rotational frequency $f_{r}$. Table 4 summarizes these frequencies as functions of $f_{r}$ and were used to identify BPFO and BSF components in the envelope spectra shown in Figure 5 and Figure 6.

The observed spectral peaks in Figure 5 and Figure 6 coincided with the theoretically predicted bearing fault frequencies and their harmonics, confirming that the applied envelope analysis successfully demodulates bearing-induced impulsive responses and enables the reliable extraction of fault-related spectral features.

### 3.3. Feature Matrix Construction

Following the extraction of time-domain, frequency-domain, and physics-informed features, the next step consists of organizing these features into a structured matrix suitable for supervised learning.

In the MaFaulDa dataset, each measurement consists of six vibration signals (three axes on the underhang bearing and three axes on the overhang bearing) and a tachometer reference signal. After feature computation, each measurement is represented by a 192-dimensional feature vector, corresponding to 32 features per axis across six axes. Consequently, the final dataset is organized as a matrix of size N×192, where N denotes the total number of measurements after preprocessing and class balancing.

Table 6 summarizes the extracted feature groups, the number of features per group, the signal representations from which they are derived, and the associated signal processing techniques.

### 3.4. Normalization of Extracted Features

All extracted features were scaled using min–max normalization to the range [0, 1] prior to model training. This normalization prevents features with larger numerical ranges from dominating the learning process and ensures numerical stability during classifier training.

Min–max normalization is applied to each feature independently according to>

(8) $$x_{\mathrm{norm}}=\frac{x-x_{\min}}{x_{\max}-x_{\min}}\;$$

where

$x_{\mathrm{norm}}$ denotes the normalized feature value,
x is the original feature value,
$x_{max}$ is the maximum value of the given feature over the dataset, and
$x_{min}$ is the minimum value of the given feature over the dataset.

This normalization ensures that all features contribute comparably to the learning process while preserving their relative variations and fault-related information.

The normalized feature matrix obtained through the procedures described above was used as the input to the supervised machine learning algorithms, whose training and testing are presented in the next section.

## 4. Training and Testing of Diagnostic Models

This section describes the development, training, and testing of supervised machine learning models for vibration-based fault diagnosis. The proposed diagnostic system employs multiple classifiers and a two-layer decision strategy in order to identify the dominant fault type and assess the presence of imbalance as a coexisting fault. Model performance was evaluated using cross-validation and a set of complementary performance metrics.

### 4.1. Supervised Learning Algorithms

The diagnostic models were based on supervised machine learning algorithms, where each feature vector was associated with a known fault label. Three widely used classifiers were considered: Support Vector Machines (SVM), Random Forest (RF), and Gradient Boosting (GB). These algorithms were selected due to their proven effectiveness in vibration-based fault diagnosis and their complementary learning characteristics.

In addition to the individual classifiers, an ensemble model based on weighted soft voting was constructed by combining SVM, RF, and GB. This approach exploits the different decision mechanisms of the classifiers: geometric separation in SVM, stochastic decision aggregation in Random Forest, and additive error correction in Gradient Boosting. The ensemble employs weighted soft voting, where the weights assigned to the individual classifiers are determined through a discrete grid search. Integer weight combinations in the set {1, 2, 3} were evaluated for each classifier, and the configuration achieving the highest mean 10-fold cross-validation accuracy was selected.

### 4.2. Training and Testing

Model training and testing were performed using a 10-fold cross-validation strategy. The extracted feature vectors were randomly partitioned into ten subsets in a way that in each iteration, nine subsets were used for training and one for testing, until all subsets had served as test data once. This procedure provides a reliable estimate of generalization performance and reduces sensitivity to a specific data split.

To ensure reproducibility of the experiments, a fixed random seed (42) was used for all data partitioning procedures. Hyperparameter optimization was conducted for each classifier using grid-based search within the predefined parameter ranges. The optimal hyperparameter sets corresponded to those yielding the highest cross-validation accuracy.

### 4.3. Performance Evaluation Metrics

Model performance was evaluated using four complementary metrics: overall accuracy, precision, recall, and F1-score. For multiclass classification, macro-averaging was applied so that each fault class contributed equally to the final metric values, regardless of class size. This is particularly important in diagnostic applications, where all fault types are considered equally critical.

In addition to numerical metrics, confusion matrices were used to analyze misclassification patterns across fault classes. The confusion matrices were normalized by rows, such that each true class summed 100%, ensuring equal importance of all fault classes.

### 4.4. First Layer: Multiclass Fault Classification

The first layer of the diagnostic model performs multiclass classification to identify the dominant machine condition. Two feature configurations were evaluated:

(i) purely data-driven feature set, and
(ii) hybrid feature set combining data-driven and physics-informed features.

Table 7 summarizes the classification performance of SVM, RF, GB, and the weighted soft-voting ensemble for both feature configurations after hyperparameter optimization, using features extracted from all six measurement axes (three axes on the overhang bearing and three axes on the underhang bearing). Table 7 also lists the optimal hyperparameter values obtained through cross-validation for each model.

For the ensemble model, voting weights were determined through a discrete grid search procedure. Specifically, integer weight combinations in the set {1, 2, 3} were evaluated for each classifier, resulting in 27 possible weight triplets. For each configuration, the mean 10-fold cross-validation accuracy was computed, and the weight combination yielding the highest accuracy was selected. For the data-driven configuration, the optimal weight combination was [2, 1, 1], resulting in an accuracy improvement of 1.743 percentage points compared to the best individual classifier. For the hybrid configuration, the optimal weight combination was [1, 2, 1], yielding an improvement of 0.769 percentage points over the strongest standalone model.

The results demonstrate a consistent improvement when physics-informed features are incorporated. For all considered classifiers, the hybrid models outperformed the purely data-driven models in terms of accuracy, precision, recall, and F1-score. Among all of the evaluated models, the ensemble classifier trained with hybrid features achieved the highest overall performance.

**Figure 7:**
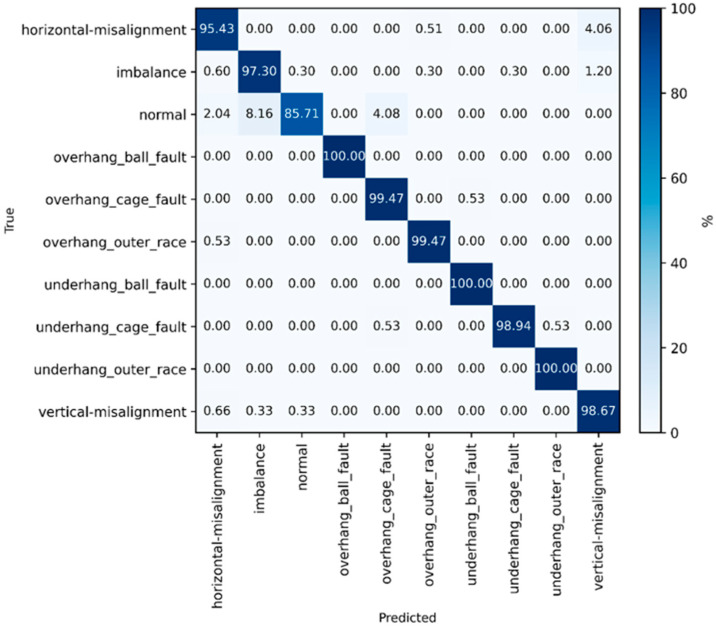
Row-normalized confusion matrix for multiclass fault classification obtained with the selected hybrid ensemble model using features from all six measurement axes (three axes on each bearing).

To further analyze class-wise diagnostic performance, the confusion matrix of the selected hybrid ensemble model using all six measurement axes is shown in Figure 7. The matrix was row-normalized so that each row summed 100%, allowing for a direct comparison of recall across fault classes. The results indicate high recognition rates for all fault types, with only limited confusion between mechanically related conditions.

Based on these results, the ensemble algorithm trained with a hybrid feature set was selected for further analysis.

Using the selected combination of an algorithm and type of feature set, an additional study was conducted to investigate the influence of measurement axis selection on diagnostic performance. The ensemble classifier was therefore trained using features extracted from individual axes and from different combinations of axes. The corresponding performance values are reported in Table 8.

The results show that models based on single-axis measurements already provide competitive performance, particularly for tangential and radial directions. However, combining multiple axes consistently improved the classification accuracy. Although the highest performance was obtained when all six axes from both bearings were used, such a configuration is rarely available in practical condition monitoring systems, where measurements are often limited to one or two sensor directions.

#### Feature Importance Analysis

To quantitatively assess which features drive the performance improvement of the hybrid feature set and evaluate the contribution of physics-informed features, a permutation-based feature importance analysis was conducted for the selected hybrid ensemble model. The importance of each feature was estimated as the mean decrease in accuracy after random permutation using the cross-validation evaluation method.

**Figure 8:**
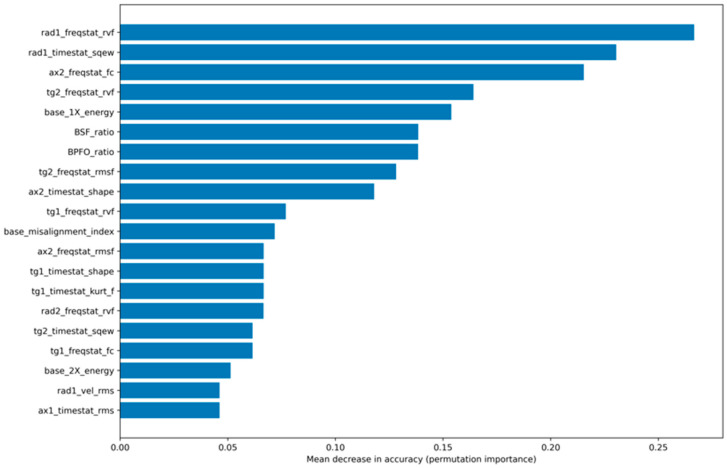
Top 20 features ranked by permutation importance (mean decrease in cross-validated accuracy) for the hybrid ensemble model using all six measurement axes.

Figure 8 presents the top 20 most influential features ranked according to their mean permutation importance. The results indicate that both frequency-domain and time-domain statistical features contributed significantly to the model performance. Physics-informed features such as energy contained at the first harmonic, BSF_ratio, BPFO_ratio, and the misalignment index ranked among the most influential features, demonstrating that physically meaningful frequency-band energy indicators actively contribute to classification.

To further quantify the relative contribution of different feature groups, the cumulative permutation importance was aggregated by feature category (frequency-domain, time-domain, and physics-informed features). The results are summarized in Table 9. Frequency-domain features accounted for 42.46% of the total importance, time-domain features for 33.15%, and physics-informed descriptors contributed 24.39% of the overall model importance.

The importance distribution does not indicate reliance on a single dominant feature, but rather reflects a balanced contribution from multiple complementary descriptors. Notably, physics-informed features accounted for approximately one-quarter of the total model importance, providing complementary diagnostic information that enhances classification robustness under varying operating conditions. These findings confirm that the performance improvement observed with the hybrid feature set is not incidental but is directly associated with the integration of physically interpretable fault indicators.

### 4.5. Second Layer: Binary Imbalance Detection

The second layer of the diagnostic system performs binary classification to detect the presence of imbalance as a coexisting fault. The dataset was reorganized into two classes:

○ class 0—no imbalance, and
○ class 1—imbalance present.

Since the resulting class distribution was approximately balanced, no additional class balancing procedure was required. The combination of an ensemble algorithm and hybrid feature set that was selected from the multiclass classification task was reused for this binary classification problem. Model performance was evaluated using the same 10-fold cross-validation protocol, and overall accuracy was used as the primary evaluation metric.

Table 10 reports the binary classification accuracy obtained for different axis configurations.

The results confirm that the proposed approach is effective for imbalance detection. As in the multiclass case, multi-axis configurations consistently outperformed single-axis setups, demonstrating the benefit of sensor fusion for detecting coexisting faults.

The outcome of this section is a trained two-layer diagnostic model that first identifies the dominant fault class and subsequently evaluates the presence of imbalance as an additional defect. In the following section, the selected combination of a ensemble algorithm and hybrid feature set is validated using a previously unseen dataset measured in experiments in order to assess its robustness, generalization, and practical applicability.

## 5. Validation of Diagnostic Models

This section validates the proposed diagnostic framework and the selected best-performing hybrid ensemble models on previously unseen vibration signals that were not used during training. The objective was to assess the generalization capability and transferability of the diagnostic framework under measurement conditions that differed from the training dataset, including differences in the bearing type, sensor configuration, acquisition hardware, and operating speed.

Following the results reported in Section 4, validation was performed using the two-layer diagnostic strategy:

(i) Layer 1 (multiclass classification) identifies the dominant machine condition (fault type), and
(ii) Layer 2 (binary classification) detects the presence of imbalance as a coexisting fault (imbalance absent/present).

Since practical industrial monitoring systems often provide measurements from a limited number of sensor directions, validation focused on the hybrid ensemble model trained using the tangential and radial directions on the overhang bearing (tg1 + rad1), which achieved high cross-validation performance while reflecting a realistic and frequently used measurement setup.

### 5.1. Experimental Test Rig and Measurement System

**Figure 9:**
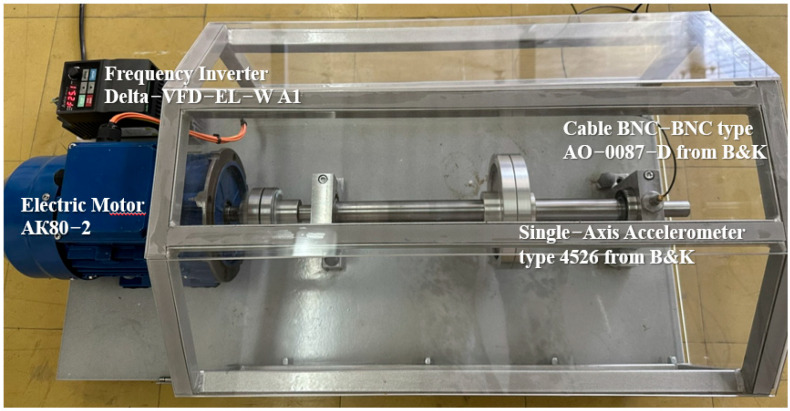
Experimental rotating machine simulator with mounted single-axis accelerometer on the overhang bearing housing.

The experimental validation was performed on a rotating machine test rig that was designed by the authors and manufactured for the purposes of this study. The experiments were conducted in the Laboratory for Vibration and Noise at the Faculty of Mechanical Engineering—Skopje. As shown in Figure 9, the rig consisted of an electric motor, a shaft, bearing supports, and a frequency inverter enabling controlled variation of the rotational speed. Bearings of type 6205-2RS were installed on the shaft and replaced according to the investigated fault condition.

**Figure 10:**
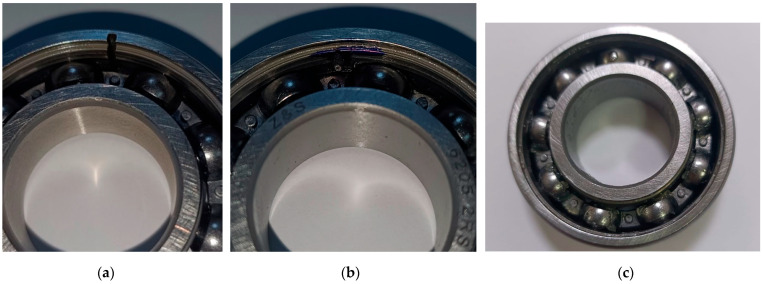
Examples of damaged bearings used for validation: (**a**) localized outer race defect; (**b**) pitting on the outer race; (**c**) combined cage and ball defect.

To generate reproducible fault scenarios, bearing defects were intentionally introduced for this study using an electric discharge machining (EDM) process with a shaped electrode, allowing for precise and repeatable creation of localized outer race defects, pitting damage, and combined cage and ball faults. Figure 10 illustrates examples of the manufactured bearing defects used in the validation experiments.

**Figure 11:**
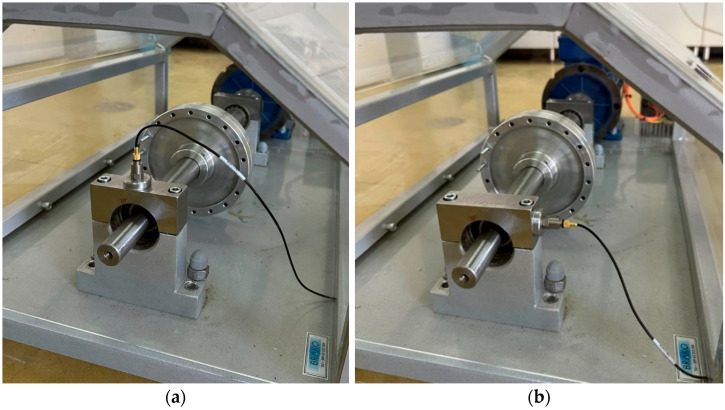
Placement of the single-axis accelerometer on the overhang bearing housing in the (**a**) radial and (**b**) tangential directions.

Vibration signals were acquired using a single-axis accelerometer (type 4526, Brüel & Kjær) mounted on the housing of the overhang bearing by means of a magnetic base (Figure 9 and Figure 11). To capture vibration components associated with different fault excitation directions, the accelerometer was mounted sequentially in two orthogonal orientations:

(i) radial direction, perpendicular to the shaft axis in the vertical direction, and
(ii) tangential direction, perpendicular to the shaft axis in the horizontal direction, as illustrated in Figure 11. This configuration enables the evaluation of diagnostic performance under realistic sensor placement conditions commonly used in industrial vibration monitoring.

**Figure 12:**
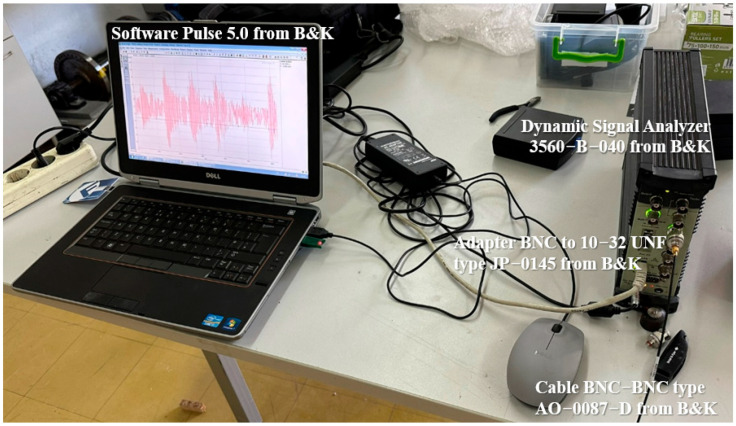
Measurement system used for vibration signal acquisition.

The accelerometer was connected to a dynamic signal analyzer (type 3560-B-040, Brüel & Kjær), and data were recorded using Pulse 5.0 software (Figure 12). The sampling frequency was set to 10 kHz, providing sufficient bandwidth for bearing fault analysis and envelope spectrum extraction.

Measurements were performed at two shaft rotational frequencies, 25 Hz and 50 Hz, corresponding to low- and medium-speed operating regimes. The dataset included signals recorded under normal operation, imbalance conditions, localized outer race defect, surface pitting on the outer race, combined ball and cage defect, and combined fault cases where imbalance coexists with a bearing defect. A detailed description of the experimental signals, including operating speed, measurement direction, and true fault condition for each validation sample, is provided in Appendix A.

### 5.2. Signal Processing and Feature Extraction

The vibration signals recorded on the experimental test rig were processed using the same signal processing and feature extraction pipeline described in Section 3, in order to ensure full methodological consistency between the training and validation phases.

For each measured signal, four signal representations were considered:

(i) the time-domain vibration signal,
(ii) the frequency-domain spectrum,
(iii) the baseband spectrum, and
(iv) the envelope spectrum.

From each signal representation, the same set of data-driven and physics-informed features used during model training was extracted (Table 6). All extracted features were subsequently normalized using the same min–max normalization scheme applied in the training phase. This step ensures numerical compatibility between the training and validation data and prevents scale differences from influencing the classifier outputs.

As a result, each validation signal was represented by a feature vector with the same structure and dimensionality as the training data, enabling direct application of the previously trained diagnostic models without any retraining or parameter adjustment.

Based on the geometry of the validation bearing (Table 11), and the applied shaft speeds, the characteristic fault frequencies (BPFO, BSF and FTF) were recomputed using the standard bearing kinematic relations shown in Equations (5)–(7). For the validation experiments conducted at 25 Hz and 50 Hz, the expected fault frequencies were approximately BPFO = 89.3/178.7 Hz, BSF = 58.0/116.1 Hz and FTF = 9.9/19.9 Hz, respectively. These frequencies and their harmonics were used to interpret the baseband and envelope spectra and identify fault-related spectral components.

**Figure 13:**
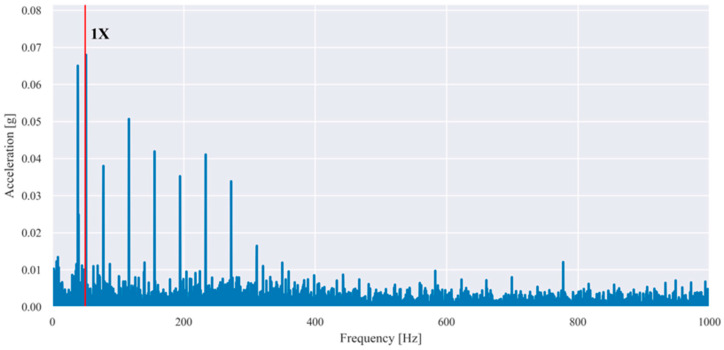
Baseband spectrum of the vibration signal measured in the tangential direction at 50 Hz shaft speed in the presence of an imbalance of 10 g (1× harmonic is dominant and highlighted with red).

**Figure 14:**
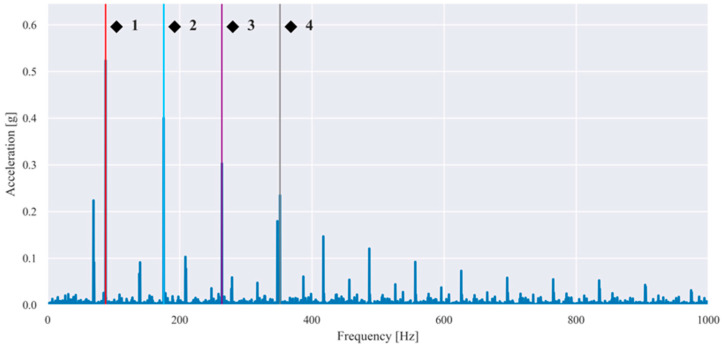
Envelope spectrum of the vibration signal measured in the radial direction at 25 Hz shaft speed for a localized outer race defect in the presence of an imbalance of 10 g. (BPFO and its 2×, 3×, 4× harmonics are highlighted in red (◆^1^), blue (◆^2^), purple (◆^3^), and grey (◆^4^).

**Figure 15:**
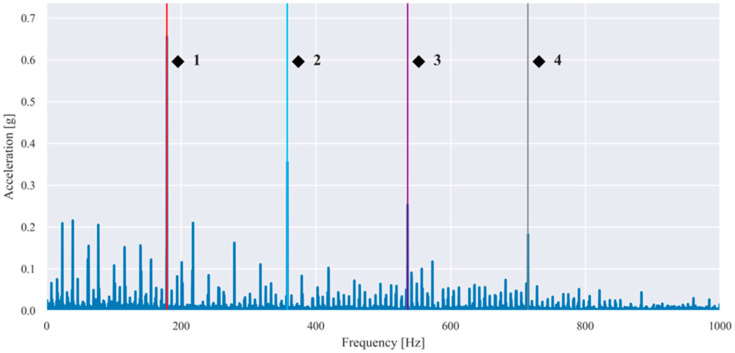
Envelope spectrum of the vibration signal measured in the tangential direction at 50 Hz shaft speed for an outer race pitting fault in the presence of an imbalance of 10 g. (BPFO and its 2×, 3×, 4× harmonics are highlighted in red (◆^1^), blue (◆^2^), purple (◆^3^), and grey (◆^4^).

Representative examples of baseband and envelope spectra obtained from the validation dataset are shown in Figure 13, Figure 14 and Figure 15 for selected fault conditions.

### 5.3. Results and Discussion

Table 12 summarizes the validation results obtained by applying the selected hybrid ensemble model (trained using tangential and radial directions on the overhang bearing, tg1 + rad1) to the experimental dataset. For each measured signal, the table reports the true machine condition, the predicted dominant fault class (Layer 1), the predicted imbalance state (Layer 2), and the corresponding vibration level expressed in terms of RMS velocity.

Out of the 36 validation signals, the proposed two-layer diagnostic system correctly classified 24 cases, corresponding to an overall validation accuracy of 66.67% when both the dominant fault class and the imbalance state were considered jointly. For signals containing simultaneous cage and ball defects, a prediction of either cage fault or ball fault was considered correct at the dominant fault level, since both defects belong to the same bearing fault family and generate overlapping spectral signatures. This value was lower than the cross-validation accuracy obtained on the training dataset (96.348 ± 0.831 for the hybrid ensemble model along the tg1 + rad1 axes), which is expected due to the differences between the training and validation conditions, including bearing type, fault geometry, sensor configuration, acquisition system, and operating environment. These factors introduce a domain shift that challenges the generalization capability of the models and provides a realistic assessment of their robustness.

The results indicate that imbalance conditions are generally well-detected by the second-layer binary classifier. Most signals recorded under imbalance conditions (both 5 g and 10 g) were correctly identified as “imbalance present”, particularly at higher vibration levels. Misclassifications of imbalance mainly occurred for low-severity imbalance cases (5 g), where vibration amplitudes remained close to those of normal operation, leading to partial overlap in the feature space.

Regarding dominant fault classification, the model demonstrated good discrimination between normal operation and severe fault conditions, as well as between imbalance and bearing-related faults. However, confusion was observed among bearing fault subclasses, particularly between outer-race defects, ball faults, and cage faults. This behavior can be attributed to the similarity of their spectral signatures in the envelope and baseband spectra, especially under the presence of coexisting imbalance, which tends to dominate the vibration response and mask localized defect components. From a practical perspective, such confusion is less critical, since all of these subclasses indicate bearing degradation requiring corrective action.

Notably, several misclassified cases corresponded to combined fault scenarios (bearing defect with simultaneous imbalance) where the model correctly identified the presence of imbalance but assigned an incorrect dominant fault type. This observation confirms the benefit of the two-layer diagnostic strategy: even when the dominant fault class is misidentified, the model remains capable of detecting imbalance as an additional coexisting defect, which is highly relevant for maintenance decision-making.

The MaFaulDa dataset has been extensively used in vibration-based fault diagnosis research, and several studies have reported classification performance using subsets of the same database. Table 13 summarizes representative prior works and compares them with the proposed approach.

Lopez [69] developed supervised classifiers using 19 statistical input features and evaluated Random Forest and Support Vector Machine models for three operating conditions (normal, imbalance, and misalignment), reporting test accuracies of 100% and 99.5%, respectively, under an 80/20 evaluation protocol. Martins et al. [70] investigated Random Forest classifiers using 19 and 31 statistical features for three-class and six-class classification problems, reporting 94.3% and 97.5% accuracy, respectively, under the same evaluation strategy. Martins et al. [71] extended the analysis to imbalance severity classification and achieved 94.14% accuracy under threefold cross-validation. Additional studies such as Ribeiro et al. [72], Marins et al. [73], and Rocha [74] reported accuracies between approximately 92% and 99% depending on the feature set size and evaluation protocol. Ince et al. [75] and Zhang et al. [76] reported classification accuracies exceeding 97–99% using convolutional neural network (CNN) architectures such as 1D CNN and WDCNN using the Case Western Reserve University (CWRU) dataset. They performed training and testing on the same dataset. Spadini et al. [77] employed acoustic features and gradient boosting models, reporting high cross-validation accuracy under controlled experimental settings.

**Table 13:** Comparison of related studies on MaFaulda and CWRU datasets.

Study	Dataset	Number and Type of Features	Classes	Model	Reported Accuracy
[69]	MaFaulDa (subset)	19 statistical features	3 classes (normal, imbalance, misalignment)	Random Forest/SVM	100%/99.5% (80/20 split)
[70]	MaFaulDa (subset)	19/31 statistical features	3 classes (normal, imbalance, misalignment)/6 classes (normal, imbalance, horizontal misalignment, vertical misalignment, underhang bearing defect, overhang bearing defect)	Random Forest	94.3%/97.5% (80/20 split)
[71]	MaFaulDa (subset)	31 statistical features	3 classes (three imbalance severity levels)	Random Forest	94.14% (3-fold CV)
[75]	Motor current dataset (own dataset)	/	2 classes (healthy, faulty)	1D CNN	97.4% (10-fold CV)
[76]	CWRU	/	10 bearing fault classes	WDCNN (Deep CNN)	>97%
[72]	MaFaulDa/CWRU	44 features: statistical (time & frequency domain) + spectral features	6 classes (MaFaulDa)/Bearing faults (CWRU, not specified)	Similarity-Based Modeling + Random Forest	96.43% (MaFaulDa) /98.7% (CWRU)
[73]	MaFaulDa/CWRU	44 features: statistical (time & frequency domain) + spectral features	6 classes (MaFaulDa)/4 bearing faults (CWRU)	Similarity-Based Modeling + Random Forest	98.5% (MaFaulDa)/98.9% (CWRU)
[74]	MaFaulDa	36 statistical features	6 classes (normal, imbalance, horizontal misalignment, vertical misalignment, underhang bearing defect, overhang bearing defect)	KNN/SVM/XGBoost	91.8%/96.9%/98.7%
[77]	MaFaulDa (acoustic only, imbalanced)	50 features: Time-domain, frequency-domain, MFCC + deltas, statistical acoustic features	42 classes (fault type + severity)	XGBoost (boosted trees)	97.90% (cross-validation, 8 kHz/8-bit)
[78]	CWRU	Downsampled vibration signals + STFT spectrogram	12 bearing fault classes	Lite CNN	~99%
[79]	MaFaulDa	/	5 classes (normal, imbalance, horizontal misalignment, bearing cage fault, bearing outer race fault)	PdM-CNN (Conv1D) + compression	High 90% range
[80]	CWRU/TMFD/MaFaulDa	/	Binary (healthy/faulty) + Multi-class (6 classes—MaFaulDa/10 classes—CWRU/3 classes—TMFD)	Hybrid RBSO–MRFO Optimized Transformer-LSTM	CWRU: 99.72% (binary & multi); MaFaulDa: 99.98% (binary), 98.60% (multi); TMFD: 99.97%
Proposed Method (This Work)	MaFaulDa (full balanced dataset)	32 hybrid features per axis 192 hybrid features for six axes (data-based and physics-informed)	Multi-class + binary (two-layer strategy)	Hybrid Ensemble	>90% (single/dual axis) 98.31% (six axes) (10-fold CV)

In comparison, the present study evaluated an expanded hybrid feature representation consisting of 32 features per axis, or 192 descriptors per six axes, combining statistical and physics-informed features extracted from multiple signal domains. Furthermore, unlike most prior works that focused exclusively on single-layer classification, the proposed framework implemented a two-layer diagnostic strategy capable of detecting coexisting faults (dominant fault type + imbalance presence).

The proposed framework explicitly evaluates the accuracy in cross-domain generalization on an independent experimental setup. However, the independent validation dataset consisted of 36 signals, which represents a limited sample size for statistical generalization. With 24 correctly classified signals, the observed joint two-layer accuracy of 66.67% corresponded to 24/36 correct predictions. Under a binomial assumption, the estimated 95% confidence interval for the true accuracy was approximately ±15 percentage points.

This implies that a change in a single prediction would shift the reported accuracy by approximately 2.8 percentage points. Therefore, while the obtained results demonstrated the transferability of the proposed framework to a new experimental setup, they should be interpreted as indicative rather than conclusive. Expanding the validation dataset to include additional operating speeds, imbalance levels, and repeated measurements per condition would further strengthen statistical reliability and represents an important direction for future work.

To partially mitigate the limited sample size and further assess model robustness, an additional noise robustness evaluation was conducted, as presented in the next section. In this extended analysis, additive white Gaussian noise (AWGN) was injected at multiple SNR levels to simulate increasingly adverse industrial conditions. This procedure effectively increased the diversity and variability of the validation data by evaluating model stability under controlled noise perturbations, thereby providing complementary evidence regarding generalization behavior.

### 5.4. Noise Robustness Evaluation

To address the robustness of the proposed diagnostic framework under realistic industrial conditions, additional validation was performed by artificially injecting additive white Gaussian noise (AWGN) into the validation signals. This procedure was conducted in response to the observation that laboratory datasets are typically cleaner than vibration signals recorded in industrial environments, where background noise and structural interferences are often significant.

**Figure 16:**
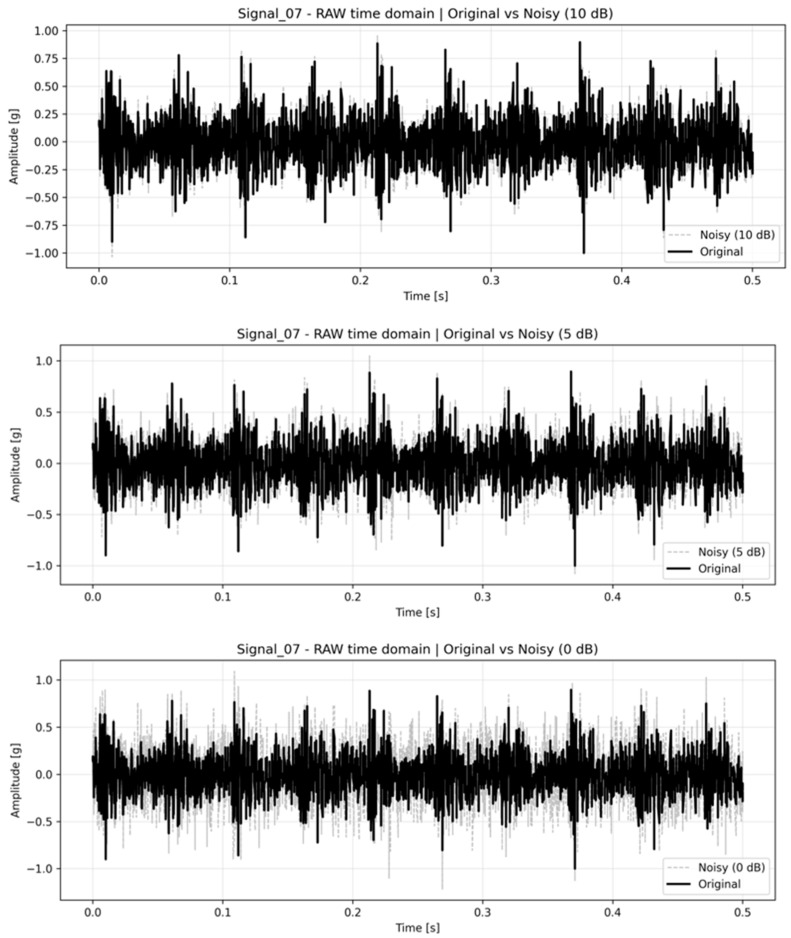

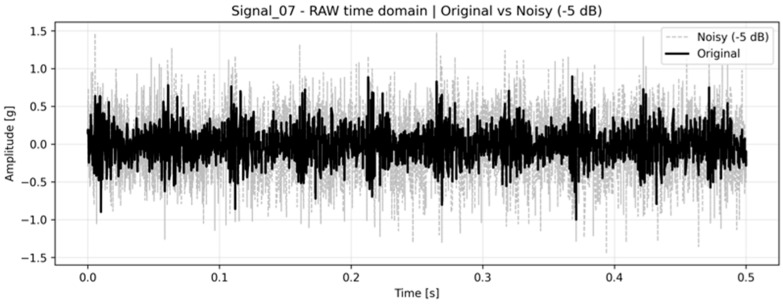
Time-domain representation of the original signal and its noisy versions at Low SNR levels.

Noise was added at multiple signal-to-noise ratio (SNR) levels: 40, 30, 20, 15, 10, 5, 0, and −5 dB. For each SNR level, the same feature extraction and two-layer classification procedure described in Section 3 and Section 4 was applied without retraining the models. Baseline results shown in Section 5.3 correspond to the measured signals without artificial noise injection. Figure 16 presents the time plots of the original (Signal_35) and noisy signals with SNR levels of 10, 5, 0, and −5 dB.

**Figure 17:**
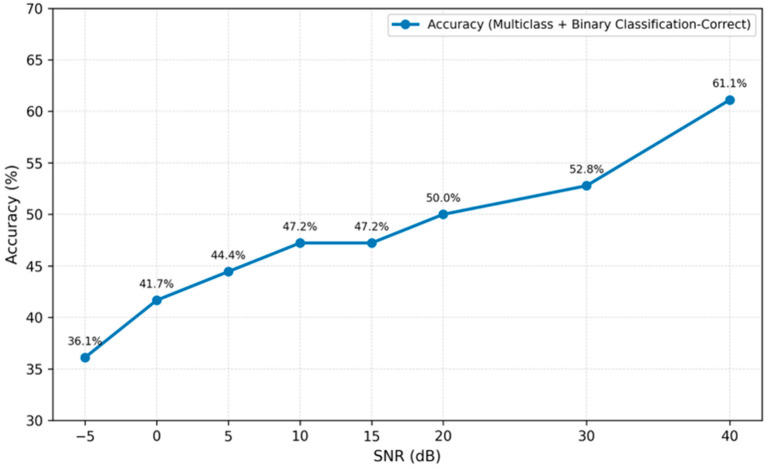
Classification accuracy of the proposed framework under additive white Gaussian noise with varying SNR levels.

Figure 17 presents the overall validation accuracy obtained when both the dominant multiclass prediction (Layer 1) and the imbalance detection result (Layer 2) were required to be correct simultaneously.

The results demonstrated a gradual and monotonic degradation of classification performance as noise intensity increased. Importantly, the framework maintained performance significantly above the random baseline even at 0 dB SNR, where signal and noise powers were equal.

Performance remained relatively stable down to 10 dB SNR, indicating resilience to moderate noise levels typically encountered in industrial monitoring systems. Severe degradation was observed only under extreme noise conditions (≤0 dB), which represent highly adverse environments rarely encountered in properly installed vibration monitoring systems.

These findings confirm that the integration of physics-informed features with statistical descriptors enhances robustness under noisy conditions, supporting the suitability of the proposed framework for industrial applications.

### 5.5. Computational Cost and Industrial Applicability

The proposed diagnostic framework is intended for deployment in industrial condition monitoring environments, where computational efficiency represents a critical practical requirement. In addition to classification performance, the computational cost associated with feature extraction and model inference was therefore systematically evaluated.

All experiments were performed on a workstation equipped with an Intel^®^ Core™ i7-10750H CPU (6 cores, 12 threads, base frequency 2.60 GHz) and 16 GB DDR4 RAM (2933 MHz), running Windows 10 64-bit. The implementation was carried out in Python 3.11.1, using the scikit-learn 1.3.0 library and NumPy 1.25.2. This configuration represents a standard mid-range workstation and provides a reproducible baseline for the reported results. 

The computational time required for extraction of the full feature set (192 features) from the training dataset was 17 min 24.72 s for 2147 segmented signal windows (after balancing the MaFaulDa dataset), corresponding to an average processing time of 486.595 ms per signal window.

For completeness, training and testing times were also evaluated for each of the algorithms that built the ensemble model using the full feature set of 192 features, as summarized in Table 14. Since the ensemble classifier was implemented as a soft voting scheme combining the already trained base models, it did not introduce additional training overhead beyond the individual model training times.

Since model training is performed offline and the trained models are subsequently stored for deployment, the training time does not affect real-time applicability. Once trained, the models are directly used for prediction.

The inference time of the trained classifiers was measured by evaluating the average prediction time per signal window after feature extraction. For the selected hybrid ensemble model using all six measurement axes (192 features), the mean inference time was 18.456 ms per signal window. Single-axis configurations exhibited inference times between 17.79 ms and 32.47 ms per signal window.

By combining feature extraction and inference times, the total processing time per signal window was approximately:

486.595 ms + 18.456 ms ≈ 505 ms per signal window.

This corresponds to a theoretical processing throughput of approximately 2 processed signal windows per second under the tested hardware configuration.

It should be noted that real-time feasibility depends on the relationship between processing time and signal window duration. In typical industrial vibration monitoring systems, signals are segmented into windows of 1–2 s duration. Since the total processing time (≈0.505 s) is lower than the acquisition time of a 1 s window, the proposed framework satisfies real-time processing requirements under standard monitoring configurations.

While deep learning-based approaches reported in the recent literature often achieve high diagnostic accuracy, they frequently require GPU acceleration and increased computational resources for training and deployment, particularly when using large-scale architectures. In contrast, the proposed framework operates using CPU-only computation and maintains sub-second processing per signal window, making it suitable for industrial edge deployment scenarios where computational resources may be limited.

To sum up, the hybrid ensemble model achieved 98.31% accuracy using signals from six axes under 10-fold cross-validation on the balanced MaFaulDa dataset. This performance is comparable to the upper range of classical machine learning approaches reported in the literature. Importantly, the contribution of the present study extends beyond absolute accuracy values. The framework explicitly evaluates:

- Cross-domain generalization on an independent experimental setup,
- Statistical uncertainty associated with limited validation samples,
- Robustness under additive white Gaussian noise down to −5 dB SNR,
- Computational efficiency and real-time feasibility.

Despite the limited number of validation signals, the study remains valuable because it evaluates cross-domain generalization across different bearing types, measurement hardware, and rotational speeds, introducing a realistic domain shift scenario not typically addressed in benchmark-based studies.

Many benchmark studies primarily optimize performance within a single dataset under controlled laboratory conditions. In contrast, the present work emphasizes robustness, interpretability, and industrial applicability alongside competitive classification performance.

Therefore, the proposed hybrid feature-based framework provides a balanced trade-off between diagnostic accuracy, physical interpretability, computational efficiency, and robustness under adverse operating conditions.

Furthermore, the vibration severity levels, expressed in terms of RMS velocity, were consistent with the predicted machine conditions. Signals classified as imbalance or combined faults exhibited substantially higher RMS values compared to normal and isolated bearing fault cases, supporting the physical plausibility of the predictions and their alignment with standard vibration-based condition assessment practices. In this context, the vibration levels can be directly interpreted using international standards such as ISO 10816-1 [81] and ISO 20816-1 [82], enabling the assessment of fault severity and the urgency of maintenance actions.

Overall, the validation results demonstrate that the proposed diagnostic framework can be transferred from a laboratory dataset to an independent experimental setup with moderate but practically meaningful accuracy under different operating and measurement conditions. The observed performance degradation highlights the inherent difficulty of cross-domain fault diagnosis and underscores the importance of hybrid feature sets and multi-stage decision strategies for improving robustness. These findings confirm that the proposed approach provides a practical and interpretable diagnostic solution suitable for real-world vibration monitoring scenarios, where perfect matching between training and operational data cannot be assumed.

## 6. Conclusions and Future Work

This study presented a supervised machine learning-based diagnostic framework for vibration-based fault diagnosis of rotating machinery using integrated data-driven and physics-informed feature sets. Multiple signal representations, including time-domain, frequency-domain, baseband, and envelope spectra, were processed in parallel to extract complementary information associated with different fault mechanisms. The extracted features were evaluated using Support Vector Machines, Random Forests, Gradient Boosting, and a weighted ensemble classifier within a two-layer diagnostic strategy.

The first layer identifies the dominant fault type and the second layer evaluates the presence of imbalance as a coexisting fault. Comparative analysis between purely data-driven and hybrid feature sets showed that the inclusion of physics-informed features significantly enhanced diagnostic performance across all evaluated classifiers. Among the tested models, the hybrid ensemble classifier achieved the best overall cross-validation results, confirming the benefit of combining statistical descriptors with features linked to physical fault signatures.

An additional analysis of measurement axis selection showed that although single-axis models already provide competitive performance, combining multiple axes consistently improves diagnostic accuracy. However, the results also indicated that high performance can be achieved using only tangential and radial measurements on a single bearing, which corresponds to a realistic sensor configuration in practical condition monitoring systems.

The proposed diagnostic framework was validated using an independent experimental dataset acquired on a different test rig with a different bearing type, sensor configuration, and operating speeds. The validation results demonstrated that the model can be transferred from the training dataset to previously unseen experimental conditions with moderate but practically meaningful accuracy. The observed performance reduction compared to the cross-validation results reflects the inherent difficulty of generalizing data-driven models across different machines and measurement environments. Nevertheless, the two-layer strategy proved advantageous, particularly in combined fault scenarios, as the model was able to detect imbalance even when the dominant bearing fault type was not perfectly identified.

In addition, the vibration severity levels expressed in terms of RMS velocity were consistent with the predicted machine conditions and aligned with international vibration evaluation standards. This enables the direct interpretation of diagnostic outputs in terms of fault severity and maintenance urgency, supporting practical decision-making in condition monitoring applications.

Although deep learning approaches have attracted significant attention in recent rotating machinery diagnosis research due to their automatic feature learning capabilities and high classification performance on large raw datasets, they typically require extensive labelled data, substantial computational resources, and offer limited interpretability in contrast to classical methods. The present study demonstrates that a hybrid feature engineering strategy, combined with classical supervised learning models, can achieve robust, accurate fault classification with efficient training and physically interpretable feature representation. Therefore, despite the advancements of deep learning, feature-based machine learning approaches remain highly relevant for practical fault diagnosis tasks, particularly in industrial environments characterized by moderate dataset sizes and real-time constraints. Future work will include systematic benchmarking against deep learning architectures to further quantify these performance trade-offs.

Overall, the results confirm that integrating data-driven and physics-informed features within a multi-stage supervised learning framework improves robustness, interpretability, and the practical applicability of vibration-based fault diagnosis. The proposed approach offers a flexible and extensible solution suitable for industrial condition monitoring applications, where measurement conditions, sensor placement, and machine configuration may vary.

Although the independent validation dataset consisted of 36 signals, which limits statistical generalization, the associated uncertainty was explicitly quantified using a binomial confidence interval (±15% at 95% confidence). To further assess robustness under more adverse operating conditions, an additional noise robustness evaluation was conducted by injecting additive white Gaussian noise at multiple SNR levels down to −5 dB. The results demonstrated gradual performance degradation with increasing noise intensity while maintaining meaningful accuracy at moderate SNR levels. These findings provide complementary evidence of model stability beyond the limited physical validation dataset.

Future work will focus on extending the experimental validation to larger and more diverse datasets, including additional operating speeds, multiple imbalance levels, repeated measurements per condition, and data acquired in industrial environments, in order to further improve statistical confidence and generalization performance. As previously stated, deep learning-based models will be investigated and compared with the proposed hybrid feature-based approach in order to assess their potential for automatic feature learning. The diagnostic framework will also be extended to include more diverse fault types and additional coexisting fault types and more complex fault combinations, enabling more comprehensive condition monitoring under realistic operating conditions.

## Figures and Tables

**Table 1:** Technical specifications of the rolling bearings in SpectraQuest Machinery Fault Simulator in creating the MaFaulda dataset [63].

Parameter	Value	Unit
Number of balls	8	/
Ball diameter $D_{B}$	7.145	mm
Cage diameter $D_{C}$	28.519	mm
Pitch diameter ($D_{P}=D_{C}-D_{B}$)	21.374	mm
Contact angle φ	41.5	°

**Table 2:** Example of extracted time-domain statistical features for both bearings and all three axes in the presence of a ball defect on the overhang bearing and an imbalance of 6 g at a rotational speed of 39.73 Hz.

Axis	RMS	Std	Kurtosis	Skewness	Peak Value	Crest Factor	Impulse Factor	Margin Factor	Shape Factor	Entropy	v_RMS_ [mm/s]
rad1	1.421	0.958	3.181	0.212	11.710	4.419	5.557	6.553	1.258	2.995	1.909
rad2	0.247	0.158	3.735	−0.810	1.554	3.693	4.803	5.764	1.301	3.199	2.276
tg1	0.660	0.446	3.045	0.117	5.053	3.990	5.003	5.907	1.254	3.071	1.613
tg2	1.651	1.112	3.009	0.096	13.164	4.142	5.200	6.149	1.255	3.031	1.633
ax1	2.080	1.443	2.952	−0.466	13.242	3.702	4.582	5.337	1.238	3.229	1.306
ax2	2.064	1.203	5.466	−1.311	14.128	4.518	6.267	7.754	1.387	3.044	1.424

**Table 3:** Example values of frequency-domain statistical features for the three measurement axes on both bearings, under a ball defect in the outer bearing and an imbalance level of 6 g at a shaft rotational speed of 39.73 Hz.

Axis	FC	RMSF	RVF
rad1	1227.873	77.938	41,888.840
rad2	488.238	51.764	31,723.491
tg1	1383.045	129.652	38,653.991
tg2	1079.394	47.671	61,450.099
ax1	1055.075	109.448	34,399.185
ax2	546.829	53.874	40,722.829

**Table 4:** Example values of phase-based spectral features for the three measurement axes on both bearings, under a ball defect in the outer bearing and an imbalance of 6 g at a shaft rotational speed of 39.73 Hz.

Axis	Phase Angle φ [rad]	sin (φ)	cos (φ)
rad1	2.200	0.808	−0.589
rad2	2.400	0.675	−0.737
tg1	−2.700	−0.428	−0.904
tg2	−2.000	−0.909	−0.416
ax1	0.500	0.480	0.878
ax2	2.000	0.909	−0.416

**Table 5:** Characteristic frequencies of rolling bearing defects expressed as functions of the rotational frequency $f_{r}$.

Defect Type	Characteristic Frequency
FTF	0.3750 $f_{r}$
BPFO	2.9980∙$f_{r}$
BSF	1.402∙$f_{r}$

**Table 6:** Overview of the extracted feature groups, signal representations, and processing methods. The bold values are the number of features and the values that are not in bold are the values that indicate the number of axes.

Feature Type	Feature Group	Number of Features	Signal Representation	Processing Method
Time-domain	Statistical features (RMS, Std, Kurtosis, Skewness, Peak, Crest, Impulse, Margin, Shape, Entropy, v_RMS_)	**11** × 6 = **66**	Prepared time-domain signal	None (directly from the prepared time signal)
Frequency-domain	Statistical features (RMSF, FC, RVF)	**3** × 6 =**18** **3** × 6 =**18**	Amplitude spectrum of prepared signal	FFT of prepared signal
	Spectral Features (phase angle φ and sinφ and cosφ)			
Physics-informed	Energy at rotational harmonics ($E_{1X},\;{MIS}_{index}$)	**2** × 6 = **12**	Baseband spectrum	Baseband analysis + FFT
	Energy around bearing characteristic frequencies ($BPFO_{ratio},\;{BSF}_{ratio},\;{FTF}_{ratio})$ and their harmonics $\left(E_{BPFO\_1X}-E_{BPFO\_4X}\right)$ $\left(E_{BSF\_1X},\;E_{BSF\_2X}\right)$ $\left(E_{FTF_{1X}}-\;E_{FTF_{4X}}\right)$	**3** × 6 + **10** × 6 = **78**	Envelope spectrum	Band-pass + Hilbert + FFT
**Total per axis 32** **Total (3 axes on two bearings) 192**				

**Table 7:** Performance of models using data-driven and hybrid feature sets for multiclass classification using all six measurement axes (three axes on each bearing) after hyperparameter optimization with 10-fold cross-validation.

Feature Type	Algorithm	Accuracy ± Std [%]	Precision ± Std [%]	Recall ± Std [%]	F1-Score ± Std [%]	Optimal Hyperparameter Values
Data-Driven	SVM	89.851 ± 1.133	88.129 ± 1.051	87.755 ± 1.157	87.937 ± 1.112	C = 50; γ = 0.05
	RF	89.363 ± 1.103	88.015± 1.153	87.592 ± 1.189	87.002 ± 1.164	n_estimators = 400; max_depth = None
	GB	89.036 ± 1.258	87.328 ± 1.205	86.503 ± 1.333	86.914 ± 1.283	n_estimators = 250; lr = 0.05; depth = 3
	Ensemble	91.594 ± 0.954	89.981 ± 1.001	89.491 ± 1.034	89.558 ± 0.991	weights = [2, 1, 1]
Hybrid (Data-Driven + Physics Informed)	SVM	94.618 ± 0.852	94.350 ± 0.814	94.127± 0.894	94.247 ± 0.859	C = 10; γ = 0.01
	RF	97.539 ± 0.588	97.021 ± 0.605	96.751 ± 0.644	96.881 ± 0.626	n_estimators = 200; max_depth = 20
	GB	96.770 ± 0.665	95.469 ± 0.732	95.460 ± 0.718	95.657 ± 0.713	n_estimators = 250; lr = 0.1; depth = 3
	Ensemble	98.308 ± 0.236	97.853 ± 0.198	97.499 ± 0.266	97.679 ± 0.219	weights = [1, 2, 1]

**Table 8:** Multiclass classification performance of the selected hybrid ensemble model for different measurement axis configurations (10-fold cross-validation).

Axis/Axes	Accuracy ± Std [%]	Precision ± Std [%]	Recall ± Std [%]	F1-Score ± Std [%]	Ensemble Weights
ax1	86.982 ± 1.652	82.624 ± 2.086	80.696 ± 2.017	80.863 ± 2.446	[1, 2, 1]
tg1	94.514 ± 1.828	95.143 ± 2.190	92.157 ± 2.319	93.222 ± 2.061	[1, 2, 1]
rad1	93.796 ± 1.596	94.319 ± 2.136	91.652 ± 2.530	92.619 ± 2.334	[1, 2, 1]
ax2	84.265 ± 2.581	81.853 ± 2.128	79.686 ± 2.089	80.226 ± 2.505	[1, 2, 1]
tg2	93.491 ± 1.976	94.085 ± 1.772	91.539 ± 2.070	92.226 ± 2.584	[1, 2, 1]
rad2	92.055 ± 1.877	92.518 ± 2.669	91.953 ± 2.716	91.955 ± 2.682	[1, 2, 1]
ax1 + ax2	89.133 ± 1.948	89.755 ± 2.385	88.356 ± 2.942	88.529 ± 2.757	[1, 2, 1]
tg1 + tg2	94.110 ± 1.001	94.208 ± 1.457	93.832 ± 1.903	94.013 ± 1.604	[1, 2, 1]
rad1 + rad2	95.843 ± 1.026	94.912 ± 1.347	93.667 ± 1.875	94.283 ± 1.483	[1, 2, 1]
tg1 + rad1	96.353 ± 0.951	95.955 ± 1.209	95.698 ± 1.462	95.823 ± 1.303	[1, 2, 1]
tg2 + rad2	96.036 ± 1.023	95.035 ± 1.303	94.605 ± 1.574	94.997 ± 1.458	[1, 2, 1]
ax1 + tg1 + rad1	96.874 ± 0.326	96.961 ± 0.344	96.582 ± 0.355	96.615 ± 0.379	[1, 2, 1]
ax2 + tg2 + rad2	96.668 ± 0.355	96.848 ± 0.346	95.774 ± 0.381	96.084 ± 0.387	[1, 2, 1]
All axes on both bearings	98.308 ± 0.236	97.853 ± 0.198	97.499 ± 0.266	97.679 ± 0.219	[1, 2, 1]

**Table 9:** Cumulative permutation importance aggregated by feature group for the hybrid ensemble model.

Feature Group	Cumulative Importance	Contribution [%]
Frequency-domain	1.2409	42.46
Time-domain	0.9688	33.15
Physics-informed	0.7127	24.39

**Table 10:** Binary classification performance of the selected hybrid ensemble model for imbalance detection under different measurement axis configurations (10-fold cross-validation).

Axis/Axes	Accuracy ± Std [%]	Ensemble Weights
ax1	87.453 ± 1.153	[1, 2, 1]
tg1	94.541 ± 1.056	[1, 2, 1]
rad1	94.601 ± 0.981	[1, 2, 1]
ax2	91.671 ± 1.203	[1, 2, 1]
tg2	94.349 ± 1.003	[1, 2, 1]
rad2	93.057 ± 1.008	[1, 2, 1]
ax1 + ax2	91.889 ± 0.954	[1, 2, 1]
tg1 + tg2	95.874 ± 0.883	[1, 2, 1]
rad1 + rad2	96.425 ± 0.852	[1, 2, 1]
tg1 + rad1	96.348 ± 0.831	[1, 2, 1]
tg2 + rad2	96.326 ± 0.829	[1, 2, 1]
ax1 + tg1 + rad1	97.424 ± 0.486	[1, 2, 1]
ax2 + tg2 + rad2	97.333 ± 0.471	[1, 2, 1]
All axes on both bearings	98.777 ± 0.309	[1, 2, 1]

**Table 11:** Technical specifications of the rolling bearings type 6205 2RS used in the experimental rig.

Parameter	Value	Unit
Number of balls	9	/
Ball diameter $D_{B}$	7.94	mm
Cage diameter $D_{C}$	46.44	mm
Pitch diameter ($D_{P}=D_{C}-D_{B}$)	38.5	mm
Contact angle φ	0	°

**Table 12:** Validation results of the proposed two-layer diagnostic model using the hybrid ensemble classifier (tg1 + rad1 configuration).

Signal ID	True Condition	Predicted Dominant Condition	Predicted Imbalance Condition	Result	V_RMS_ [mm/s]
Signal_01	Normal	Normal	Imbalance Absent	Correct	0.152
Signal_02		Normal	Imbalance Absent	Correct	0.195
Signal_03		Normal	Imbalance Absent	Correct	0.106
Signal_04		Normal	Imbalance Absent	Correct	0.183
Signal_05	Imbalance	Normal	Imbalance Absent	Incorrect	0.518
Signal_06		Normal	Imbalance Present	Incorrect	0.419
Signal_07		Normal	Imbalance Present	Incorrect	0.427
Signal_08		Imbalance	Imbalance Present	Correct	0.750
Signal_09		Imbalance	Imbalance Present	Correct	4.450
Signal_10		Imbalance	Imbalance Present	Correct	2.997
Signal_11		Imbalance	Imbalance Present	Correct	4.974
Signal_12		Imbalance	Imbalance Present	Correct	3.375
Signal_13	Outer race localized defect on overhang bearing	Underhang outer race	Imbalance Absent	Incorrect	3.351
Signal_14		Overhang outer race	Imbalance Absent	Correct	3.390
Signal_15		Overhang outer race	Imbalance Absent	Correct	3.280
Signal_16		Overhang ball fault	Imbalance Absent	Incorrect	3.483
Signal_17	Outer race localized defect on overhang bearing + imbalance	Overhang ball fault	Imbalance Present	Incorrect	4.313
Signal_18		Overhang outer race	Imbalance Present	Correct	4.318
Signal_19		Overhang outer race	Imbalance Present	Correct	4.538
Signal_20		Overhang outer race	Imbalance Present	Correct	4.765
Signal_21	Outer race pitting on overhang bearing	Overhang ball fault	Imbalance Absent	Incorrect	3.296
Signal_22		Overhang ball fault	Imbalance Absent	Incorrect	3.412
Signal_23		Overhang outer race	Imbalance Absent	Correct	3.304
Signal_24		Overhang outer race	Imbalance Absent	Correct	3.340
Signal_25	Outer race pitting on overhang bearing + imbalance	Overhang cage fault	Imbalance Present	Incorrect	4.200
Signal_26		Overhang outer race	Imbalance Present	Correct	4.234
Signal_27		Underhang outer race	Imbalance Present	Incorrect	4.155
Signal_28		Underhang outer race	Imbalance Absent	Incorrect	4.211
Signal_29	Cage + ball defect on overhang bearing	Overhang cage fault	Imbalance Absent	Correct	3.830
Signal_30		Overhang ball fault	Imbalance Absent	Correct	3.940
Signal_31		Underhang ball fault	Imbalance Absent	Incorrect	3.765
Signal_32		Overhang ball fault	Imbalance Absent	Correct	3.778
Signal_33	Cage + ball defect on overhang bearing + imbalance	Overhang cage fault	Imbalance Present	Correct	3.972
Signal_34		Overhang ball fault	Imbalance Present	Correct	3.963
Signal_35		Overhang ball fault	Imbalance Present	Correct	4.325
Signal_36		Overhang ball fault	Imbalance Present	Correct	4.414

**Table 14:** Training and testing times were also evaluated for each of the algorithms that built the ensemble model using the full feature set of 192 features.

Model	Training + Testing Time
SVM	102.01 s
RF	94.87 s
Gradient Boosting	3354.19 s

## Data Availability

The raw data supporting the conclusions of this article will be made available by the authors on request.

## Appendix A. Description of Validation Signals

**Table A1:** Overview of the validation signals and measurement conditions.

Signal ID	Rotation Frequency [Hz]	Axis	True Condition
Signal_01	25	vertical (rad1)	Normal
Signal_02	50	vertical (rad1)	
Signal_03	25	horizontal (tg1)	
Signal_04	50	horizontal (tg1)	
Signal_05	25	vertical (rad1)	Imbalance 5 g
Signal_06	50	vertical (rad1)	
Signal_07	25	horizontal (tg1)	
Signal_08	50	horizontal (tg1)	
Signal_09	25	vertical (rad1)	Imbalance 10 g
Signal_10	50	vertical (rad1)	
Signal_11	25	horizontal (tg1)	
Signal_12	50	horizontal (tg1)	
Signal_13	25	vertical (rad1)	Outer race localized defect on overhang bearing
Signal_14	50	vertical (rad1)	
Signal_15	25	horizontal (tg1)	
Signal_16	50	horizontal (tg1)	
Signal_17	25	vertical (rad1)	Outer race localized defect on overhang bearing + imbalance 10 g
Signal_18	50	vertical (rad1)	
Signal_19	25	horizontal (tg1)	
Signal_20	50	horizontal (tg1)	
Signal_21	25	vertical (rad1)	Outer race pitting on overhang bearing
Signal_22	50	vertical (rad1)	
Signal_23	25	horizontal (tg1)	
Signal_24	50	horizontal (tg1)	
Signal_25	25	vertical (rad1)	Outer race pitting on overhang bearing + imbalance 10 g
Signal_26	50	vertical (rad1)	
Signal_27	25	horizontal (tg1)	
Signal_28	50	horizontal (tg1)	
Signal_29	25	vertical (rad1)	Cage + ball defect on overhang bearing
Signal_30	50	vertical (rad1)	
Signal_31	25	horizontal (tg1)	
Signal_32	50	horizontal (tg1)	
Signal_33	25	vertical (rad1)	Cage + ball defect on overhang bearing + imbalance 10 g
Signal_34	50	vertical (rad1)	
Signal_35	25	horizontal (tg1)	
Signal_36	50	horizontal (tg1)	
